# Plastic, nutrition and pollution; relationships between ingested plastic and metal concentrations in the livers of two *Pachyptila* seabirds

**DOI:** 10.1038/s41598-020-75024-6

**Published:** 2020-10-22

**Authors:** Lauren Roman, Farzana Kastury, Sophie Petit, Rina Aleman, Chris Wilcox, Britta Denise Hardesty, Mark A. Hindell

**Affiliations:** 1 CSIRO Oceans and Atmosphere, Hobart, TAS Australia; 2 Institute for Marine and Antarctic Studies, University of Tasmania, Hobart, TAS Australia; 3 Future Industries Institute, University of South Australia, Adelaide, SA Australia; 4 ScaRCE Research Centre, UniSA STEM, University of South Australia, Adelaide, SA Australia; 5 Antarctic Climate and Ecosystems CRC, University of Tasmania, Hobart, TAS Australia

**Keywords:** Environmental chemistry, Marine biology, Ecology, Environmental sciences, Ocean sciences

## Abstract

Naturally occurring metals and metalloids [metal(loid)s] are essential for the physiological functioning of wildlife; however, environmental contamination by metal(loid) and plastic pollutants is a health hazard. Metal(loid)s may interact with plastic in the environment and there is mixed evidence about whether plastic ingested by wildlife affects metal(loid) absorption/assimilation and concentration in the body. We examined ingested plastic and liver concentration of eleven metal(loid)s in two seabird species: fairy (*Pachyptila turtur*) and slender-billed prions (*P. belcheri*). We found significant relationships between ingested plastic and the concentrations of aluminium (Al), manganese (Mn), iron (Fe), cobalt (Co), copper (Cu) and zinc (Zn) in the liver of prions. We investigated whether the pattern of significant relationships reflected plastic-metal(loid) associations predicted in the scientific literature, including by transfer of metals from ingested plastics or malnutrition due to dietary dilution by plastics in the gut. We found some support for both associations, suggesting that ingested plastic may be connected with dietary dilution / lack of essential nutrients, especially iron, and potential transfer of zinc. We did not find a relationship between plastic and non-essential metal(loid)s, including lead. The effect of plastic was minor compared to that of dietary exposure to metal(oid)s, and small plastic loads (< 3 items) had no discernible link with metal(loid)s. This new evidence shows a relationship between plastic ingestion and liver metal(loid) concentrations in free-living wildlife.

## Introduction

Pollution of the marine environment by plastics^1^, metals and metalloids, henceforth “metal(loid)s”, is a major environmental concern^2,3^. Some metals are dietary minerals, which are essential for the physiological functioning of organisms. These ‘essential’ metals include chromium (Cr), cobalt (Co), nickel (Ni), copper (Cu), iron (Fe), manganese (Mn), zinc (Zn). Other metal(loid)s are considered non-essential for living organisms, having no known biological benefit. These ‘non-essential’ metal(loid)s include arsenic (As), cadmium (Cd) and lead (Pb)^4^, while the biological role of aluminium (Al) is still unclear. Depending on the type, speciation and concentration, both essential and non-essential metal(loid)s can be toxic to organisms^4–7^.

Metal(loid)s and plastics can adversely affect the health of marine wildlife that consumes them^8^. Plastics may interact with metal(loid)s in the ocean by concentrating them on their surfaces through sorption^9^, and concentrations of Al, Cr, Co, Ni, Fe, Cu, Zn, Cd and Pb on the surface of beached plastic can be higher than in the background environment^9–12^. Some metals, including Zn, are plastic additives and serve multiple function as flame retardants and smoke suppressants^13^. It is not clear whether ingested metal(loid)s adsorbed onto plastics are absorbed into systemic circulation from the gastro-intestinal tract and accumulate in tissues within plastic-ingesting organisms. Similarly, little research has investigated plastic-associated metal(loid) absorption and health impacts within organisms^14^. Research linking plastic ingestion and metal(loid)s in free-living marine vertebrates is in its infancy, and baselines for metal(loid) concentrations exerting adverse health effects have not yet been established. To improve the understanding of pollutant associations, sentinel species, particularly seabirds, are routinely employed as indicators for environmental health^15,16^.

Essential metals Cu, Fe, Mn, Co and Zn are regulated physiologically in seabirds, and influenced by diet and nutritional status^17,18^. Chronic reduced food intake can decrease the availability of essential metals and other micronutrients, potentially causing sub-lethal malnutrition^19^. Because of physiological requirements for flight, birds have a higher metabolic rate than many other taxa and can starve to death within days of food restriction^20^. Basal metabolic rate is even higher for small birds, some species of which are unable to fast for longer than one night in energetically demanding conditions^21^. When extreme emaciation from starvation occurs, the body exhausts stored fat and catabolises muscle and organ proteins for energy^20^. Fasting and starvation cause changes in serum, tissues and organs metal(loid) concentration as part of catabolism, but do not change the total metal(loid) load in the body (except what is lost as urine and faeces during fasting)^22^. Protein catabolism decreases muscle and organ mass and increases the concentration of some metals, including Cu and Zn, in the liver, a finding that has been observed across multiple bird taxa^22–24^. Increased concentration of liver Cu and Zn is associated with a slow loss of liver mass, which is a physiological response to starvation^20,25^.

Plastic may influence metal concentrations in tissues and organs directly if the consumed plastic acts as a vector/carrier for metals, which may be digested, absorbed, and metabolized/assimilated in tissue and organs (henceforth “transfer”). Plastics, when eaten, may also influence tissue and organ metal(loid) concentration indirectly through ‘dietary dilution’. Dietary dilution occurs when an animal with plastic in its stomach reduces its feeding frequency or eats small amounts of food compared to an animal that has not ingested plastic^26^. Dietary dilution may result from the presence of plastic, which causes a reduction in gut volume available for nutritious food^26^ and/or an effect on hunger/satiety^27^, as has been observed in sea turtles^26,28^. In seabirds, plastic ingestion has been linked to increased Cr and silver (Ag) in feathers of fledgling flesh-footed shearwaters (*Ardenna carneipes*)^29^, but no link was found for 15 other metal(loid)s examined, nor was a plastic-metal(loid) link found in muscle tissue of fledgling short-tailed shearwaters (*A. tenuirostris*)^30^. Plastic ingestion was also linked to increased Fe, Mn and Rubidium (Rb) in feathers of Bonin petrels (*Pterodroma hypoleuca*)^31^. Nutritional consequences may include effects on tissue/organ metal(loid) concentration via dietary dilution, as ingestion of non-nutritive plastic may cause reduced feeding and intake of essential dietary mineral metals, non-essential metals and potentially malnutrition^26,28^.

Non-essential metal(loid)s, including As, Cd, Pb and mercury (Hg) accumulate in the food chain to higher-order predators, making seabirds ideal study animals in which to appraise bioaccumulation and biomagnification^32–35^. Seabirds contain some of the world’s highest reported levels of non-essential metal(loid)s detected in wildlife, but whether these cause adverse health effects is unknown^17,36–38^. Sorption of non-essential Pb and Cd by plastic have been reported in the environment^35,39,40^, and plastic has been suggested as a vector for Pb and other metals^40^. Non-essential metal(loid) uptake in seabirds, especially Cd, is considered dietary^37,38^, although plastic has also been suggested as a vector^29,31^ and has been linked with increased Pb in the feathers of Bonin petrel^31^. One experimental study suggested that plastics may be responsible for between 6 and 30% of a seabird's exposure to Pb^41^. If ingested plastic and non-essential metal(loid)s are correlated in seabirds, the pathway is likely direct as non-essential metal(loid)s are not physiologically regulated, although they can be sequestered in feathers and bones^38,42^.

**Figure 1:**
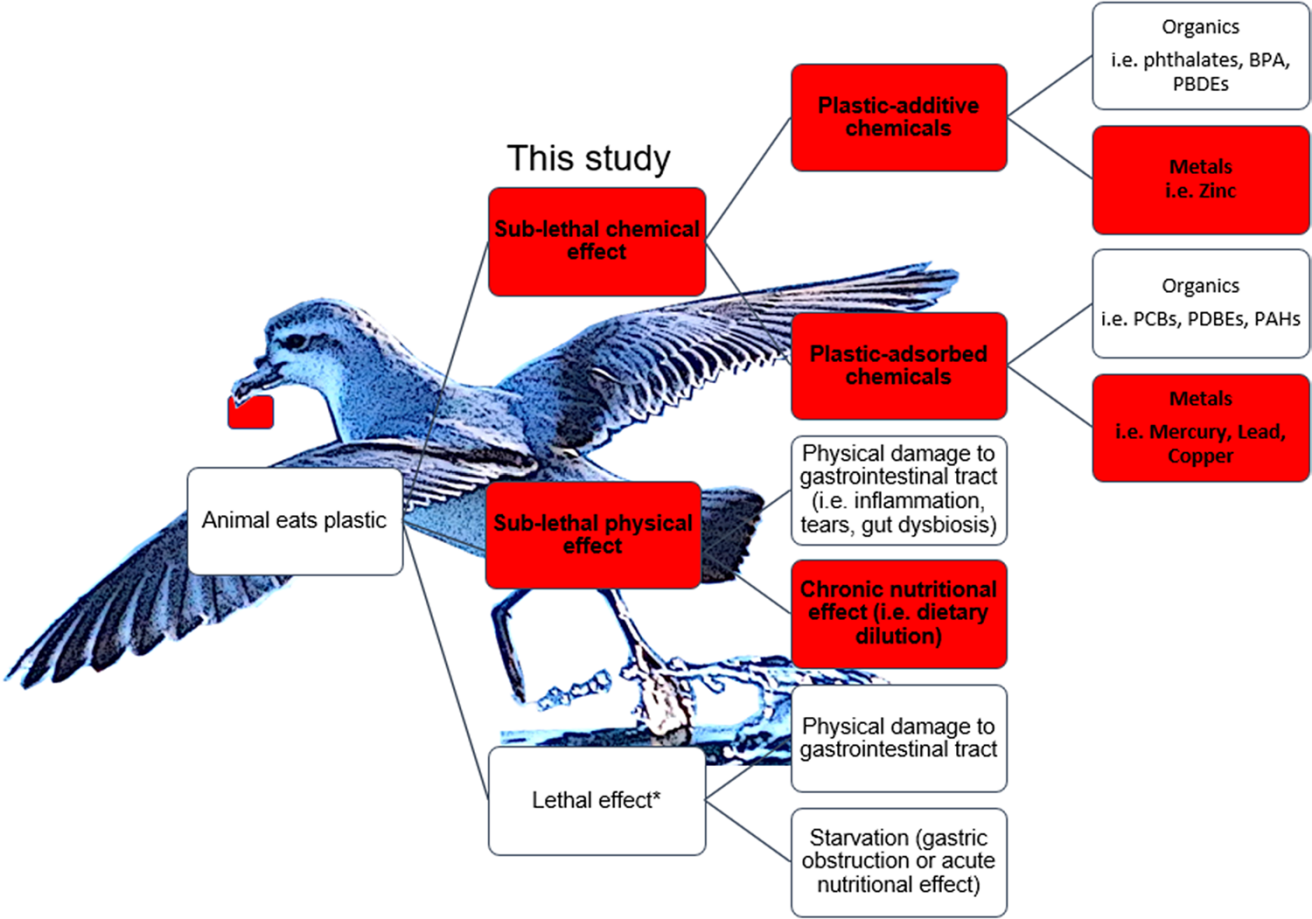
Ingestion of plastic by seabirds and other animals may lead to both lethal and sub-lethal health effects, including physical effects, linked to physical damage and nutrition, and chemical effects, concerning the transfer of plastic-additive and plastic- adsorbed chemicals. This study (red coloured boxes) explores the relationship between ingested plastic and liver metal(loid) concentration and potential associations with plastic-adsorbed metal(loid)s, plastic-additive metals and nutrition. Prion photo by L. Roman and image manipulation using GIMP^88^.

Ingestion of plastic by seabirds may lead to both lethal^43,44^ and sub-lethal health effects^45^, including physical effects, linked to physical damage and nutrition, and chemical effects, concerning the transfer of plastic-additive and plastic-adsorbed chemicals^46^ (Fig. 1). Though knowledge on the plastics and metal(loid)s in seabirds is increasing, studies exploring how plastic and metal(loid)s interact and the mechanism of significant relationships are lacking. Significant plastic-metal relationships have been found in seabird feathers^31^; plastic has been suggested as a vector for metals^40^ and plastic-nutritional effects have been found in sea-turtles^26,28^. However, metal(loid)-plastic relationships in seabird organs have never been investigated. Understanding the effects of ingested plastic on physiological factors, such as nutrition and pollution, in species of the open ocean is challenging in terms of practical and ethical concerns.

Here, we examined the relationship between metal(loid) concentration in the liver and plastic ingestion in two seabird species: fairy prion (*Pachyptila turtur*) and slender-billed prion (*P. belcheri*), opportunistically utilizing beach-cast individuals that died during significant off-shore storms in New Zealand. Fairy and slender-billed prions are small seabirds (140 g) that occur across a broad oceanic range covering much of Southern Hemisphere’s marine regions and demonstrate considerable overlap in diet and foraging ecology^47^. The plankton diet (*Euphausiid* sp. and amphipods^47^) and surface-feeding habits of prions make them vulnerable to plastic ingestion, and both species commonly ingest plastics^48^. We examined whether there was a relationship between ingested plastic and liver metal(loid) concentration in each species, as well as both *Pachyptila* species combined. A significant relationship would lead us to examine whether the association reflects positive or negative relationships predicted in the scientific literature and may result from dietary dilution or direct transfer by examining the support for each of these associative mechanisms (“Relationships in the literature and predicted associative mechanisms” section).

## Materials and methods

### Seabird sampling

Carcasses of fairy prion, *Pachyptila turtur*, (n = 26) and slender-billed prion, *P. belcheri*, (n = 25) were collected dead in emaciated condition during a *Pachyptila* “wreck” (seabird mass mortality event), on Muriwai Beach (− 36° 50′ 18″S 174°25′31."E﻿) and Karioitahi Beach (− 37°16′58″ S 174°39′14.E), in Auckland region, New Zealand. Carcasses were collected between 1–4th August 2016, following off-shore winter storms associated with high wind and rough seas from 28th July to 2nd August 2016. Average hourly wind speeds > 40 km/h, sometimes exceeding 60 km/h, were recorded at Muriwai Beach across four days between 28th and 31st July 2016, with wind and rough seas abating during the following days.

*Pachyptila* wrecks associated with winter storms are a well-known phenomenon in New Zealand^49,50^ and South America^51,52^, where persistent high wind, rain and rough seas impair/prevent prions from feeding or resting. Prolonged severe weather not only prevents feeding, but can cause an inability for birds to catabolize fuel (body fat and protein) fast enough to meet energy demands and lead to death by exhaustion and starvation, even for individuals which had initial fat reserves^53^. Tens to hundreds of thousands of prions may have died during this four-day storm event, as carcasses were observed spanning beaches across hundreds of kilometres of coastline between Muriwai, Auckland region in the North and Waikanae Beach, Wellington in the south (Lauren Roman *pers obs*). On Karioitahi Beach, Waikato/Auckland region, hundreds of prion carcasses were observed across just a few kilometres of beach (Ian Southey, *pers comm.* with Lauren Roman). Given the size of the wreck, we propose that it is unlikely that the birds involved were over-represented by unfit or poorly individuals. Carcasses were collected within two days of the storm’s abatement and plastics recovered likely reflect those eaten prior to the storm event rather than as a result of post-storm malnourishment. The carcasses were necropsied and whole livers were excised, and frozen livers exported for analysis.

### Necropsy

Carcasses were necropsied according to well-established collection and dissection procedures^54^. All wrecked seabirds showed consistent, emaciated body condition. The scored body condition for all carcasses was 0 (from range of 0–3) for each pectoral muscle, sub-cutaneous fat and intestinal fat according to seabird necropsy procedures^54^, alleviating potential confounding factor of mixed body condition. Bird age was assigned as either “immature” (including juvenile and immature) or “adult” according to gonad development, following seabird necropsy procedures^54^.

The contents of the proventriculus and gizzard were removed and visually inspected for anthropogenic debris. Stomachs were empty except for plastic, showing birds had not fed before death, and the few natural items found (squid beak, pumice) were excluded from analysis. All debris items visible to the naked eye were removed, counted and weighed using electronic scales. Livers were excised, wrapped in aluminum foil and frozen at − 20 °C until analysis.

### Metal(loid) analysis

The livers were rinsed with MilliQ water, freeze-dried (Modulyod Freeze Dryer) and pre-digested in 70% HNO_3_ (10 mL) overnight. The samples were digested using a block digester (A.I. Scientific AIM500) by slowly ramping up the temperature to a maximum of 170 °C until the organs were dissolved and the samples dried to near evaporation (~ 1–2 mL)^55^. The samples were made up to 10 mL using MilliQ water, vortexed thoroughly to mix, syringe filtered (0.45 µm) to separate the dissolved metal(loid)s and stored at 4 °C until analysis using inductively coupled plasma spectrometry (Agilent 8800), following USEPA method 6020A^56^. Eleven metal(loid)s were examined: Al, As, Cd, Cr, Mn, Fe, Co, Ni, Cu, Pb and Zn.

### Quality assurance and quality control

We used Standard Reference Material (SRM) 2976 from the National Institute of Standards and Technology during the digestion of livers. The accuracy of the digestion method was confirmed by a quantitative average ± standard error of the mean (SEM) Pb recovery of 1078 ± 14.0 mg/kg (n = 4) from SRM 2976 (certified total 1190 ± 0.18 mg/kg). During the analysis using ICP-MS, the QA/QC process outlined in the USEPA method 6020A was followed. The limit of detection (LOD) for Pb was 0.1 ppb and blanks (n = 4) were below LOD. For each element, the metal(loid) concentrations that were at least three times above its respective LOD were used in the downstream statistical analysis. The average deviation from check value recoveries (n = 17) ranged between 1.5–6.1%, indicating that the drift during analysis via ICP-MS was within the acceptable range as stipulated by USEPA method 6020A.

### Statistical analysis

To determine whether a relationship existed between plastic and liver metal(loid) concentration, we first examined whether there was a relationship between the presence of ingested plastic in the gut and metal(loid) concentration in the liver. Where a statistically significant relationship between presence of plastic and the concentration of a metal(loid) occurred, we examined whether there was a relationship between the metal(loid) concentration and the load of plastic (number of items and mass of items) inside the bird. For each plastic-metal(loid) relationship, fairy prions and slender-billed prions were tested individually, followed by both prion species (*Pachyptila* sp.) pooled together, to explore whether potential relationships are species specific or common between the two *Pachyptila* species. Statistical analysis was conducted using R 3.5.0^57^.

#### Metal(loid) concentration and plastic presence

We examined whether a statistically significant relationship existed between the presence of ingested plastic and the concentration of each of the 11 metal(loid)s in the liver. We conducted a Mann–Whitney U test for each prion species and both combined (pooled prions), testing the response variable, liver metal(loid) concentrations (mg/kg), against predictor variables of ingested plastic presence in individuals. Mann–Whitney U test was chosen because of the skewed distribution of metal(loid) concentration.

#### Metal(loid) concentration and plastic load in each species

We first tested for co-linearity between the number of ingested plastic items and ingested item mass, using "Variance Inflation Factor (VIF)" to determine whether one or both parameters are required in plastic load statistical analyses. We used the “VIF” function in the “usdm” package^58^, with a VIF value > 5 to represent co-linearity between number of ingested plastic items and plastic mass.

Where significant relationships were observed between metal(loid) concentration and plastic presence, we used generalized linear models (GLMs) to investigate the relationship between liver metal(loid) concentrations (mg/kg) and plastic loads (number of items and mass of items). We conducted a GLM for each prion species, testing the response variable, liver metal(loid) concentrations (mg/kg), against predictor variables of ingested number of items and mass of items.

#### Metal(loid) concentration and plastic load in pooled prions

We used GLMs to investigate the relationships between ingested plastic load (both number of items and plastic mass) and metal(loid) concentration where significant plastic-presence relationships were found across pooled prions, testing a unique model for each relevant metal(loid). These models included co-variates for prion species and bird age group to disentangle these potential non-plastic impacts, which may confound these plastic-metal(loid) relationships. The response variable was the concentration of each metal(loid) (mg/kg) in the liver and predictor variables were species, age, ingested plastic mass, and number of ingested plastic items. We then applied the “dredge” function in the “MuMIn” package^59^ to determine the best model using Akaike Information Criterion (AIC)^60^. We chose the model with the lowest AIC to determine whether there was a relationship between plastic load (plastic mass and/or number of pieces) and liver metal(loid) concentration (GLM including plastic mass and or number of pieces is the best model) or no relationship between plastic load and liver metal(loid) concentration. Where more than one model had ΔAIC < 2, we considered these models equivalent^61^. Means are reported with standard deviation and medians reported with 25% and 75% quartiles.

### Relationships in the literature and predicted associative mechanisms

**Figure 2:**
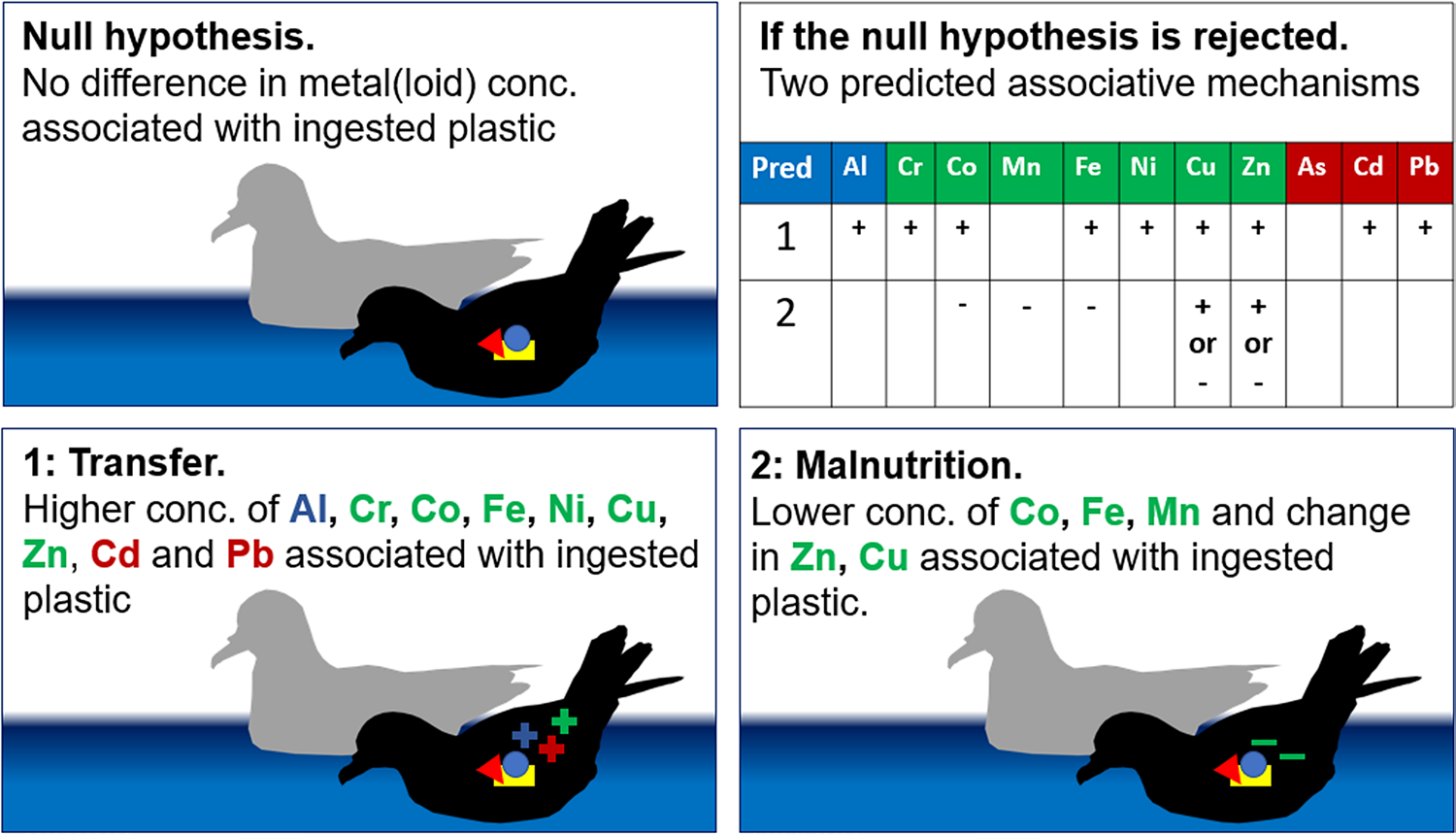
Predictions for the association of plastic with the liver metal concentration of prions, though pre-storm body condition is unknown. The null hypothesis is that there is no difference in liver metal(loid) concentration between prions with and without ingested plastic. Significant differences in the concentration of liver metal(loid)s leads to rejection of the null hypothesis and examination of support for two mechanistic association predictions derived from the literature: transfer of metal(loid)s from plastic or malnutrition due to dietary dilution by plastic in the gut. Essential metals are shown in green, non-essential metals are shown in red, and metals where the biological role is unclear are shown in blue. Prion silhouettes were created using GIMP^88^.

Where significant relationships were found between liver concentration of a metal(loid) and the presence of ingested plastic, we investigated potential associative mechanisms based on relationships predicted or suggested in the scientific literature. Two mechanisms that may link ingested plastic with organ metal(loid) concentration are found in the literature. Firstly, metal(loid)s which are plastic additives^13^ and/or adsorbed to the surface of plastic^9–12^ may transfer to the body when ingested. Secondly, malnutrition may occur as a result of dietary dilution by plastics in the gut, influencing mineral nutrient concentrations^28^. The null hypothesis was that no significant relationships existed between ingested plastic presence and liver metal(loid) concentrations, while significant relationships between plastic and liver metal(loid) would lead to two possible explanations (Fig. 2).

1. Transfer. Liver metal(loid) concentration changes may be due to transfer from plastic additive/adsorbed metals.

2. Malnutrition. Liver metal(loid) concentration changes may be due to malnutrition from dietary dilution from plastic in the gut.

Support for each transfer and malnutrition predictions are based on relationships between plastic and one or more metal(loid)s, and evidence that supports or rejects each (Fig. 2). Both or neither transfer and malnutrition explanations for significant relationships between plastic and liver metal(loid) may be supported, based on the literature, and described below. However, these associations are correlative only, so while evidence can be gathered to support each predicted mechanism, this evidence cannot prove the mechanism.

#### Evidence that supports a transfer explanation

Increases in Al, Cr, Co, Cu, Fe, Ni, Zn, Cd and Pb may be due to transfer as these metals can be adsorbed on surface of beached plastic at higher than background concentrations^9–12^ or are plastic additives (Zn)^13^, with plastic suggested in the literature as an environmental vector of Cd, Pb and other metals^29,31,40^. Therefore, a positive relationship between plastic and any of these metals would support a transfer explanation, while a negative relationship would refute a transfer explanation. A lack of relationship would neither support nor refute a transfer explanation, because not all metal(loid)s would necessarily be adsorbed onto each item of ingested plastic.

#### Evidence that supports a malnutrition explanation

Plastic ingestion is linked to decreased nutrient intake and malnourishment in sea turtles^28^, and lower concentrations of dietary minerals^17,18^ (i.e. Cu, Fe, Mn, Co and Zn) may be indicative of pre-storm malnourishment due to dietary dilution by plastics in the gut. Experimental fasting decreases hematocrit and haemoglobin in herring gulls (*Larus argentatus*)^62^, a response that can also be invoked through experimental deficiency in nutritional Fe^63^. Therefore, we expected to find low concentrations of Fe, and possibly Cu, Mn, Co and Zn^19^, as potentially indicative of pre-existing sub-lethal malnourishment. However, Cu and Zn are also known to increase with emaciation^23,24^; therefore, a an increase in Cu/Zn concentration may also support a malnutrition explanation if sub-lethally malnourished birds were more emaciated than others at the point of death (detail below in “Potential confounding factors for Copper (Cu) and Zinc (Zn)” section). A negative relationship between plastic and Fe, Mn and Co concentration would support a malnutrition explanation, and a positive relationship would refute a malnutrition explanation. A significant relationship between ingested plastic and Cu or Zn concentration, whether positive or negative, may also support a malnutrition explanation.

#### Potential confounding factors for Copper (Cu) and Zinc (Zn)

Interpreting significant change in Cu and Zn concentration poses a challenge for supporting transfer or malnutrition explanations, because two potential confounding factors may influence Cu and Zn concentration. An increase in the concentration of Cu or Zn could be due to transfer, increased emaciation before death due to malnutrition , an artefact of acute starvation in the storm and/or heavy metal detoxification. Therefore, positive relationship between Cu or Zn and plastic might support either or both transfer and malnutrition predictions or may be confounded by rapid storm starvation or heavy metal detoxification via metallothionein synthesis, because Zn and Cu concentration may be influenced by heavy metals^32,36,64,65^.

As small birds starve rapidly in storm conditions^53^, it is a challenge to disentangle acute storm starvation from other potential mechanisms, particularly sub-lethal pre-storm malnourished condition. Furthermore, little is known about sub-lethal malnourishment in wild seabirds, which adds difficulty in detecting metal(loid) signals of chronic malnourishment. When examining evidence for the malnutrition prediction, Cu and Zn may act as confounding factors since they increase with emaciation following dietary dilution / chronic malnutrition^23,24^. We expected all beach-wrecked prions examined to have higher liver concentrations of Cu and Zn than did healthy prions, as a result of protein catabolism/emaciation, but total body metal(loid) loads should not differ considerably from those of pre-storm condition. While a decrease in Cu or Zn may support the malnutrition explanation as a result of reduced dietary intake, we note that Cu and Zn are abundant in the amphipod and euphausiid crustaceans prey of prions^66^ and physiologically regulated in seabirds^17,18^. A starvation-mediated increase in Cu or Zn may also support the malnutrition explanation if seabirds that are already malnourished reach a lower final body weight before death than those in healthy pre-storm body condition, owing to death following an inability for birds to catabolize fuel (fat and protein) fast enough to meet energy demands^53^. Unfortunately, the relative weighting of a potential dietary decrease of Cu/Zu and emaciation increase of Cu/Zn cannot be tested. We posited that an increase in Cu and Zn would support the transfer explanation and a significant directional change of Cu and Zn associated with ingested plastic would support the malnutrition explanation (the direction of change may be positive or negative), while lack of change would neither support nor refute the hypotheses, but acknowledge the potential confounding effects of these metals.

### Pre-storm condition and health impact

Understanding prion body condition before their storm death poses a challenge for relating significant findings, both significant liver metal(loid)-plastic relationships and associative hypotheses, to the health of the prions. Although we do not know pre-storm body condition of prions examined, acute starvation causes body mass reduction as lipids and proteins are catabolised for energy^21,67^, but total body metal(loid) loads should not differ considerably from pre-storm condition, provided birds did not feed before death^22^. For this reason, although we can infer the influence of plastic on liver metal(loid) concentrations, we cannot test whether significant relationships lead to quantifiable health impacts.

## Results

We collected and analysed the livers of 26 fairy prions and 25 slender-billed prions. Seven fairy prions (27%) contained plastic, with a mean and standard error of 1.3 ± 0.2 pieces. Eleven slender-billed prions (44%) had ingested plastic, with a mean and standard error of 4.9 ± 1.1 pieces (Table 1). Detailed information about ingested plastic available in Supplementary Information.

**Table 1:** Summary results including ingested plastic loads of the examined fairy and slender-billed prions, median (x̃) and interquartile range (IQR) of metal(loid) concentration in the birds’ livers (mg/kg of dry mass). Concentration data for some metal(loid)s from one fairy prion were excluded.

Species	Plastic	N Bird	N Plas ± SD	Mass Plas (mg) ± SD	Al	Cr	Mn	Fe	Co	Ni	Cu	Zn	As	Cd	Pb
Fairy prion	No	19	0	0	x̃ = 251.3 IQR = 145.4–552.8	x̃ = 1.1 IQR = 1.0–2.3	x̃ = 16.6 IQR = 13.4–32.7	x̃ = 3671.2 IQR = 2414.8–4355.2	x̃ = 0.4 IQR = 0.3 – 1.0	x̃ = 1.1 IQR = 0.6–1.5	x̃ = 32.2 IQR = 26.1–46	x̃ = 210.3 IQR = 196.5–242.7	x̃ = 33.4 IQR = 21.5–42.4	x̃ = 17.1 IQR = 10.6–29.7	x̃ = 251.3 IQR = 145.4–552.8
Fairy prion	Yes	7	1.3 ± 0.5	26.0 ± 19.8	x̃ = 195.7 IQR = 56.5–365.3	x̃ = 1.2 IQR = 0.7–1.8	x̃ = 17.1 IQR = 10–28.1	x̃ = 2243.5 IQR = 1099.7–3593.9	x̃ = 0.4 IQR = 0.2–1.0	x̃ = 0.9 IQR = 0.3–1.6	x̃ = 31.8 IQR = 30–53.7	x̃ = 232.7 IQR = 198.5–293	x̃ = 30.9 IQR = 15.6–44.5	x̃ = 22.1 IQR = 13.1–37.9	x̃ = 195.7 IQR = 56.5–365.3
Fairy prion	**Total**	**26**	**0.3 ± 0.6**	**0.6**	**x̃ = 243.6 IQR = 105.8**–**506**	**x̃ = 1.1 IQR = 1.0**–**2.3**	**x̃ = 16.9 IQR = 11.3**–**33.8**	**x̃ = 3284.5 IQR = 2281.4**–**4355.3**	**x̃ = 0.4 IQR = 0.3**–**1**	**x̃ = 1.1 IQR = 0.5**–**1.6**	**x̃ = 32.0 IQR = 26.5**–**47.8**	**x̃ = 222.7 IQR = 196.1**–**266**	**x̃ = 32.1 IQR = 18.2**–**43.5**	**x̃ = 18.3 IQR = 10.8**–**30.8**	**x̃ = 243.6 IQR = 105.8**–**506**
Slender-billed prion	No	11	0	0	x̃ = 217.7 IQR = 172.2–482.2	x̃ = 1.7 IQR = 1.0–1.9	x̃ = 19.5 IQR = 12.3–37.2	x̃ = 4688.1 IQR = 2747–7175.7	x̃ = 0.4 IQR = 0.3–0.7	x̃ = 0.6 IQR = 0.4–1.1	x̃ = 40 IQR = 32.9–46	x̃ = 277 IQR = 248.1–322.8	x̃ = 23.2 IQR = 17.8–32.4	x̃ = 39.9 IQR = 33.1–55.2	x̃ = 217.7 IQR = 172.2–482.2
Slender-billed prion	Yes	14	4.9 ± 4.0	74.8 ± 108.5	x̃ = 91.7 IQR = 85–118.4	x̃ = 0.9 IQR = 0.7–1.2	x̃ = 13.3 IQR = 10.5–15.1	x̃ = 2269.9 IQR = 1625.4–3166.4	x̃ = 0.3 IQR = 0.2–0.4	x̃ = 0.8 IQR = 0.5–1.4	x̃ = 44.3 IQR = 38.3–52.3	x̃ = 329.2 IQR = 264–370.5	x̃ = 27.9 IQR = 19.8–40.7	x̃ = 63.9 IQR = 46.6–77.1	x̃ = 91.7 IQR = 85–118.4
Slender-billed prion	**Total**	**25**	**2.8 ± 3.9**	**4.9**	**x̃ = 120.5 IQR = 87.4**–**191.9**	**x̃ = 1.2 IQR = 0.7**–**1.8**	**x̃ = 13.7 IQR = 11.2**–**22.3**	**x̃ = 2880.4 IQR = 2075**–**5441.7**	**x̃ = 0.3 IQR = 0.2**–**0.5**	**x̃ = 0.7 IQR = 0.5**–**1.1**	**x̃ = 41 IQR = 35.8**–**49.8**	**x̃ = 284.3 IQR = 253.5**–**354.2**	**x̃ = 26.5 IQR = 19.1**–**38.3**	**x̃ = 61.2 IQR = 35.1**–**72.2**	**x̃ = 120.5 IQR = 87.4**–**191.9**

### Metal(loid) concentration and plastic presence

#### Fairy prion metal(loid) concentration and plastic presence

We found no significant difference (*P* > 0.05) across any of the examined liver metal(loid) concentration between plastic ingesting and non-plastic ingesting fairy prions, although Fe showed a negative trend (Table 1, U = 96, *P* = 0.094). Concentration data for some metal(loid)s from one fairy prion were excluded because of glass tube breakage.

#### Slender-billed prion metal(loid) concentration and plastic presence

**Figure 3:**
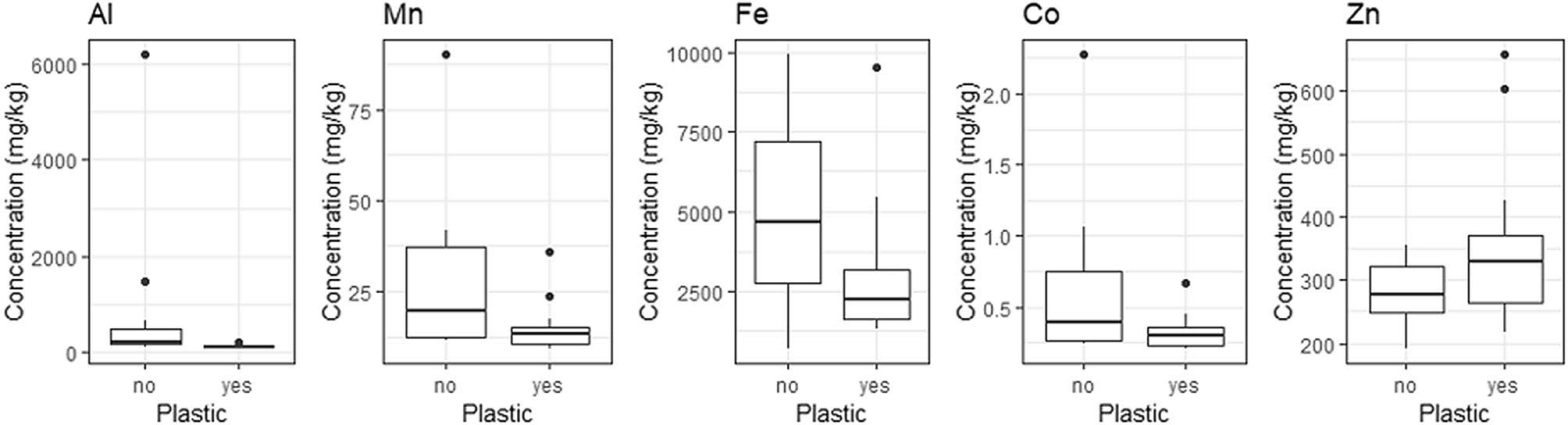
Liver concentration of Al, Mn, Fe, Co, and Zn (mg/kg of dry mass) in slender-billed prions (n = 25) showed significant differences between individuals that had not ingested (n = 11, left bars) or had ingested (n = 14, right bars) plastic at the time of death in emaciated condition during a “seabird wreck” in New Zealand, August 2016.

Slender-billed prions that had ingested plastic displayed significantly lower concentration of Al (U = 141, *P* < 0.001) and Co (U = 114, *P* = 0.048) in the liver than those that had not ingested plastic, and there was a negative trend for Mn (U = 111, *P* = 0.070) and Fe (U = 113, *P* = 0.051). In contrast, slender-billed prions that had ingested plastic had significantly higher Zn concentrations (U = 39, *P* = 0.038) (Table 1, Fig. 3).

#### Pooled prion metal(loid) concentration and plastic presence

**Figure 4:**
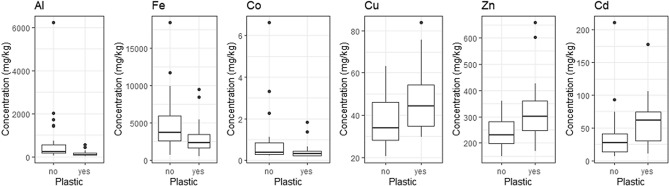
Liver concentration of Al, Fe, Co, Cu, Zn and Cd (mg/kg of dry mass) in pooled prions (n = 50), including both fairy (n = 25) and slender-billed prions (n = 25) that had not ingested (n = 30, left bars) or ingested (n = 20, right bars) plastic at the time of death in emaciated condition during a “seabird wreck” in New Zealand, August 2016.

When data from fairy and slender-billed prions were combined, pooled prions that had ingested plastic displayed significantly lower concentration of Al (U = 463, P < 0.001), Fe (U = 417, *P* = 0.020), Co (U = 405, *P* = 0.038) in the liver than those that had not ingested plastic. In contrast, pooled prions that had ingested plastic had significantly higher Cu (U = 192, P = 0.032), Zn (U = 169, *P* = 0.009) and Cd concentrations (U = 159, *P* = 0.005). In contrast to slender-billed prions, the relationship between plastic presence and Mn concentration in pooled prions was not significant (U = 379, *P* = 0.121) (Table 1, Fig. 4).

### Metal(loid) concentration and plastic load

#### Metal(loid) concentration and plastic load in slender-billed prions

For slender-billed prions, the number of ingested plastic items and mass of ingested plastic items were not colinear (VIF = 1.40), so each of these variables were tested to assess plastic load. Generalized linear models found no significant relationship between ingested plastic number nor plastic mass and Al, Mn, Fe, Co nor Zn concentrations in the liver for slender-billed prions, though the intercept value was significant for all models. Among GLMs examined, the model with the most explanatory power was the relationship between liver Fe concentration and number of ingested plastic items (X = − 202.8, Error = 135.2, R^2^ = 0.9, *P* = 0.147). Age was not included in slender-billed prion models because of lack of representation of different age groups among slender-billed prions (two adults only, see Supplementary information). Metal(loid) concentration was not assessed for fairy prions since we found no association with plastic presence (Mann–Whitney U-test results).

#### Metal(loid) concentration and plastic load in pooled prions

For pooled prions, the number of ingested plastic items and mass of ingested plastic items were not colinear (VIF = 1.53), so each of these variables were included to assess plastic load. A GLM including a predictor variable for either number or mass of ingested plastic items occurred among best equivalent models for all metal(loid)s that were found to be significantly linked to plastic presence (Al, Mn, Fe, Co and Zn), except for Cd, for which species was the only important predictor (Cd models including plastic were better than the null model but ΔAIC 2–3 from the best model) (Table 2). A model including plastic load was equivalent to a species only model for Mn, Co, Cu and Zn. A model including plastic load was better than a species only model for Al and Fe. Species appeared in top models for Fe, Co, Cu, Zn and Cd. The R^2^ for species-only models were ≤ 0.05 for most except Cu (R^2^ = 0.07), Zn (R^2^ = 0.21) and Cd (R^2^ = 0.20). Age appeared in the list of top models for Al, Mn, Fe and Co. For Al, Mn and Fe, R^2^ was higher when both age and plastic occurred in the model. For Co, R^2^ was higher for the plastic model (but less than for the species model). Age did not appear in the top models for Cu, Zn or Cd. The relationship between metal(loid) concentration and plastic was positive for Cu and Zn. The relationship between metal(loid) concentration and plastic was negative for Al, Mn, Fe and Co. R^2^ values for plastic ingestion predictor models are < 0.05 for most metals, apart from the Cu plastic + species model (R^2^ = 0.08), Zn plastic + species model (R^2^ = 0.22) and the As plastic ingestion model (R^2^ = 0.07) (Table 2).

**Table 2:** General Linear Models (GLM) and model terms describing the relationship between liver metal(loid) concentration and age, mass of ingested plastic items / number of ingested plastic items and species. Models with a difference (Δ) in AIC of < 2 are considered equivalent models, and this table shows only models within 2 AIC of the best model (except Cd, where models within 3 AIC are shown). A ‘ + ’ sign denotes inclusion of the categorical variable in the model. *Pachytila* seabirds; fairy prion (n = 26) and slender-billed prion (n = 25) were collected dead in emaciated condition during a “seabird wreck”, New Zealand 2016.

Metal	Intercept	Age	Mass plastic items	# plastic items	Species	R^2^	df	LogLik	AICc	Δ	Wgt
**Al** AIC null = 853.4	936.5	+				0.11	4	− 417.5	843.9	0	0.24
	439.3					0	2	− 420.5	845.2	1.3	0.12
	1008.6	+		− 45.57		0.13	5	− 416.9	845.2	1.3	0.12
	521.8			− 53.97		0.03	3	− 419.7	845.9	2	0.09
**Mn** AIC null = 517.8	31	+				0.11	4	− 240.3	489.5	0	0.24
	25.1					0	2	− 243.3	490.9	1.5	0.12
	27.8			− 1.76		0.03	3	− 242.5	491.5	2	0.09
	32.4	+		− 0.91		0.12	5	− 240.1	491.5	2	0.09
**Fe** AIC null = 1018	4580.5	+				0.09	4	− 482	972.8	0	0.18
	4004.3					0	2	− 484.4	973.1	0.3	0.15
	4329			− 212.31		0.04	3	− 483.4	973.4	0.6	0.13
	4158		− 6.38			0.02	3	− 484	974.5	1.7	0.07
	3909.5	+			+	0.1	5	− 481.6	974.6	1.8	0.07
	4783.1	+		− 127.99		0.1	5	− 481.6	974.6	1.8	0.07
**Co** AIC null = 167.9	0.8	+				0.1	4	− 70.4	149.6	0	0.19
	0.9				+	0.05	3	− 71.8	150.2	0.6	0.14
	0.7					0	2	− 73.1	150.5	0.9	0.12
	0.8			− 0.06		0.03	3	− 72.4	151.3	1.8	0.08
**Cu** AIC null = 530.6	36.8				+	0.07	3	− 207.3	421.2	0	0.27
	40.5					0	2	− 209.1	422.5	1.4	0.14
	39			0.97		0.04	3	− 208.1	422.8	1.6	0.12
	36.6			0.53	+	0.08	4	− 207.1	423	1.8	0.11
**Zn** AIC null = 725.4	226				+	0.21	3	− 302.7	612	0	0.43
	224.4			4.51	+	0.22	4	− 302.3	613.5	1.4	0.21
**Cd** AIC null = 565.2	27.8				+	0.2	3	− 255.2	516.9	0	0.49
	28		− 0.03		+	0.2	4	− 255.1	519.1	2.2	0.16
	27.8			− 0.04	+	0.2	4	− 255.2	519.2	2.4	0.15

### Evidence supporting associative mechanism predictions from literature

We found significant relationships between ingested plastic and seven metals. These included a significant relationship between plastic presence and Al, Mn, Fe, Co and Zn for slender-billed prions, and a significant relationship between plastic presence and Al, Fe, Co, Cu, Zn and Cd for pooled prions. However, the significant relationship between plastic presence and Cd in pooled prions was an effect of species and not of plastic (Table 2) and therefore, excluded from further inclusion.

#### Relationships supporting the transfer prediction

**Figure 5:**
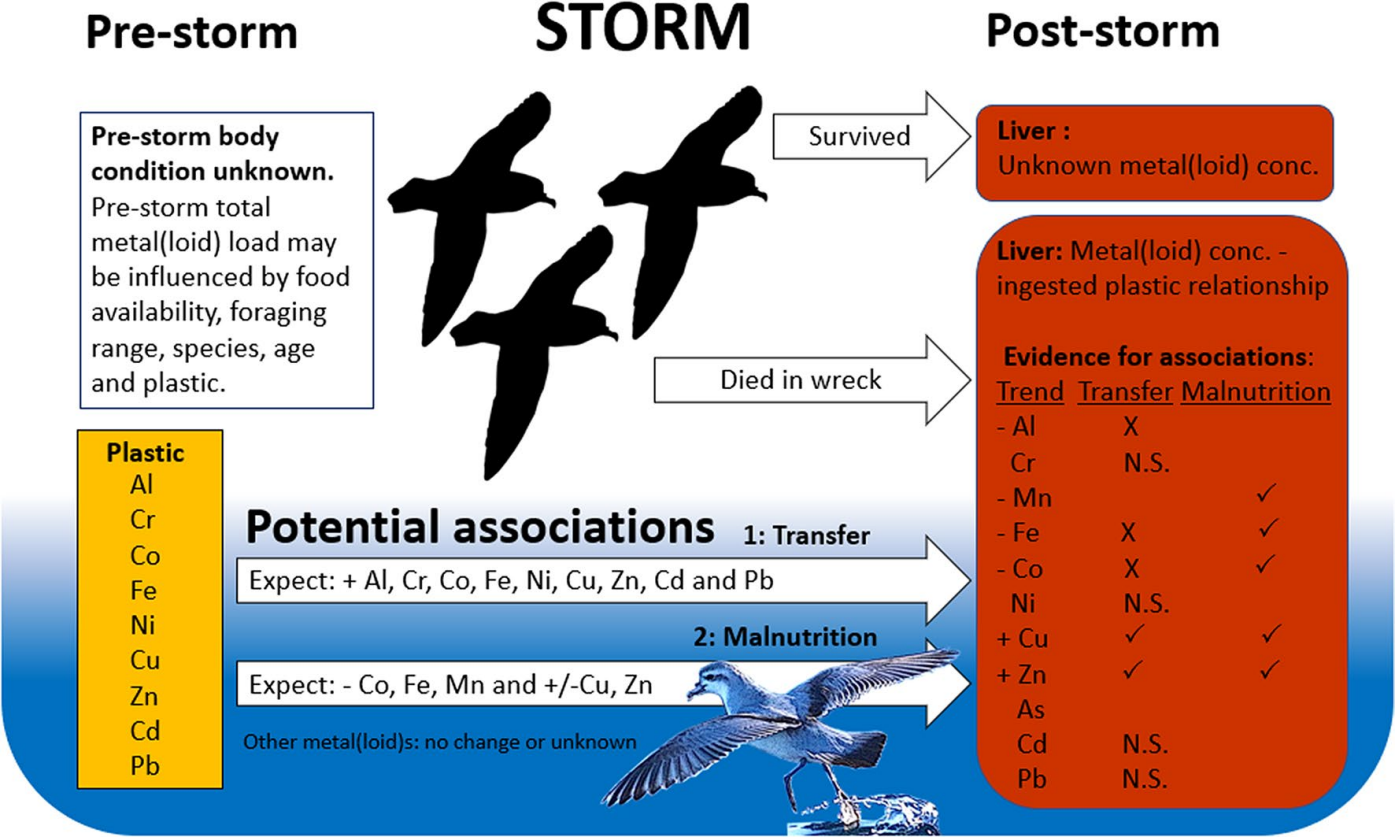
Response of liver concentration of metal(loid)s to ingested plastic in examined *Pachytila* seabirds; fairy prion (n = 25) and slender-billed prions (n = 25). Prions were collected dead in emaciated condition during a “seabird wreck”, following a significant off-shore winter storm in New Zealand, 2016, during which an estimated tens to hundreds of thousands of prions died. Statistical significance between metal(loid) concentration and plastic presence was tested by Mann–Whitney U-test. A positive sign ( +) indicates a significant positive relationship between ingested plastic and the liver concentration of each metal(loid), while a negative sign (-) indicates a significant negative relationship. Support for transfer and malnutrition associative mechanisms, predicted in the literature, is represented by a statistically significant relationship between ingested plastic and liver metal(loid) concentration. Observed relationship that match associations predicted in literature are represented by a tick (✓) and those that refute associations predicted in literature are represented by a cross (X). Not significant (N.S.) shows where a relationship was predicted, but no significant relationship was found. The results show partial support for transfer (2✓, 3X) and support for malnutrition (5✓, 0X). Prion silhouette and image manipulation created using GIMP^88^.

Significant positive relationships occurred between plastic presence and Cu (pooled prions) and Zn liver of slender-billed prions and pooled prions, which support the transfer prediction from the literature. Significant negative relationships occurred between plastic presence and Al, Fe and Co concentration in the liver of slender-billed prions and pooled prions, which refute the transfer prediction. No significant change was found for Cr, Ni, Cd or Pb. The results show partial support for the transfer prediction, with two changes in support (Cu and Zn) and three changes (Al, Fe, Cu) refuting (Fig. 5).

#### Relationships supporting the malnutrition prediction

Significant positive relationships occurred between plastic presence and Cu (pooled prions) and Zn liver of slender-billed prions and pooled prions, which support the malnutrition prediction from the literature. Significant negative relationships or trends occurred between plastic presence and Co, Mn (slender-billed prions only) and Fe concentration in the liver of slender-billed prions and pooled prions, supporting the malnutrition prediction. The significant decrease in Al associated with ingested plastic was not informative with respect to malnutrition. The results showed support for the malnutrition prediction, with five relationships in support (Cu, Zn, Co, Mn and Fe). No observed relationships refuted the malnutrition prediction (Fig. 5).

## Discussion

We found relationships between ingested plastic and liver metal concentration across six examined metals, demonstrating that ingestion of plastic is correlated with the concentration of essential metals in *Pachyptila* seabirds, although no significant link was found for non-essential metal(loid)s. Relationships between the liver metal concentrations of slender-billed prions and the presence of plastic ingestion were statistically significant, but this result did not apply to fairy prions. We found partial support for the prediction that seabirds that ingest plastic may have higher concentrations of some metals in the liver associated with transfer from plastic, and support for the prediction that ingested plastic may be associated with malnutrition (Fig. 5.), though individuals varied greatly (Table 1). We propose that the reason why we found significant effects in slender-billed but not fairy prions is that the number of ingested items is much higher in slender-billed prions (mean plastic = 4.9 ± 1.1) than in fairy prions (mean plastic = 1.3 ± 0.2). When all prions were pooled, five plastic presence-metal relationships were also significant (excluding Cd, found to be driven by species), showing that more statistical power and higher plastic loads may be required to detect significant associations between ingested plastic and liver metal concentration. A liver-metal concentration response may thus occur with larger plastic loads, though it is not detectable or is negligible for birds with small plastic loads (for example < 3 items among prions).

Results for a plastic-metal relationship in prions differ from those in short-tailed shearwaters, where no plastic-associated variation in metal concentrations in breast muscle tissue was found^30^. This may be due to inherent variation between tissues, interspecies variation, a difference in physiology and energetic requirements between free-ranging adult birds and pre-fledged altricial chicks, methodological differences, or a combination thereof. It may be that the plastic load in the gut of short-tailed shearwater chicks was too low to be associated with detectable difference in liver metal(loid) concentration for the species, similarly to fairy prions in this study. These different outcomes between short-tailed shearwater and prions suggest complexity in plastic, nutrition, and pollution relationships. Taxa should be treated individually in future studies to account for differences between species, life stage and tissue examined.

For prion liver metal concentrations, plastic-load models were better than null models for all examined metals, with plastic load or mass a predictor variable in equivalent best model for all but Cd (Table 2). This result suggests that a relationship exists between ingested plastic load and liver metal concentrations, although the biological implications of small variations are unknown. Uncertainty around the estimates of the slopes of these relationships, in view of the variation between individuals, could be reduced with increased sample sizes and better representation of large ingested plastic loads, which were underrepresented in this study. The R^2^ values of the plastic load regression models show that, irrespective of the mechanism, plastic accounts for < 10% of variation across liver metal(loid) concentrations in starving prions (Table 2). This low value indicates that plastic is likely to be playing a role, but the impact of external factors, such as diet and foraging habitat are much greater, especially where small plastic loads (< 3 items, as fairy prions) are observed. Variation due to individual factors and diet likely account for the remaining 80–95% of unexplained variation across metal(loid)s. Species had no or minimal influence on variation across most metal(loid) concentrations, except for Zn and Cd, showing the considerable overlap in liver metal(loid) concentration between fairy and slender-billed prions. These differences may be biological, or may reflect regional availability of these nutrients in amphipod and euphausiid across their differing range^47,66^. Age appeared in the top models for Al, Mn, Fe and Co, with R^2^ value of ~ 0.1 for each. This suggests that approximately 10% of the variation in these metal concentrations is related to the age of the bird, reflecting studies finding age-related variation in the liver concentration of some metal(loid)s, although patterns differ between seabird species^68,69^. For Al, Mn and Fe, age-only models have a smaller R^2^ than age-and-plastic models, showing that the effect of plastic is probably additive to age rather than confounded by it.

The malnutrition prediction was better supported than was the transfer prediction, with reduced Fe, Mn and Co indicating possible decreased intake of these dietary minerals, although the observed positive Cu and Zn relationship also supports the transfer hypotheses (Figs. 2, 5). Mixed support for the transfer predictions may reflect the transfer of only some metals from plastic to tissue, with other metals remaining bound to plastic or not present at all, confounding due to metallothionein synthesis^32,36,64^ or another mechanism that is causing the increases in Cu and Zn concentration. Zn was found in significantly higher concentrations in both slender-billed and pooled prions, while the concentration of other transfer-relevant metals did not increase significantly in the liver. Plastic-additive metals such as Zn^13^ may be a larger pollution concern than plastic-adsorbed metals, at least for marine environments with low levels of metal pollution. It is also possible that the increased Zn concentration may result from the synthesis of metallothionein, if plastic ingestion is associated with intake of heavy metals including Cd, As and mercury (Hg)^32,36,64^. Mixed support for the transfer association may also be an indicator that external and unaccounted factors are influencing these relationships; for example, birds ingesting plastic may be foraging in different locations or on different prey species. The negative Al relationship with plastic was not covered by existing literature-based predictions, indicating that other metal(loid)s may also be subject to transfer, dietary dilution, or other factors. Reduced Al may reflect dietary dilution through reduced mineral intake, similar to the relationship observed for Mn, Co and Fe. Support for these explanations are correlative only; while these correlations are meaningful in the context of the previous research which informed each association, the mechanism itself (transfer and dietary dilution) cannot be definitively tested with this observational study.

We propose that ingested plastic, especially at large loads, may influence liver metal(loid) concentration through reduced dietary intake of dietary Al, Fe, Co and Mn, dietary minerals that occur commonly in the species on which prions feed^66,70^. This reduced mineral nutrient intake may occur as a function of dietary dilution associated with larger ingested loads of ingested plastic. Plastic-mediated dietary dilution may occur over a period of a month to years of plastic retention in the gut^71,72^ causing sub-lethal chronic malnutrition, as has been observed in sea turtles^26,28^. The acute starvation preceding death in this study presents a challenge for interpreting whether the birds we sampled from the beach were in healthy or poor body condition prior to the storm that caused them to die and strand, and the possible nutritional impacts of ingested plastics as they relate to the health of the birds prior to their death.

Starvation and fasting experiments in animals show that those in ‘fat’ and ‘lean’ body condition typically have similar initial muscle protein content (except heart), while good/fat condition animals have a higher liver mass and more fat (adipose)^67^. When both ‘fat’ and ‘lean’ animals are starved, although fat animals survive for longer by metabolising fat and conserving protein (at least in the initial stages), ultimately, the limiting factor for survival is the cumulative loss of body proteins rather than lipid (fat) availability^67^. This ‘protein conservation’ differs between fat and lean animals^67^, and is affected by energy expenditure during fasting^73,74^. Energy expenditure is an important factor in this study, as very high energy expenditure during fasting would be expected in the days preceding the death of the small (~ 140 g healthy mass) birds in this study, enduring four days of storm conditions at sea^53^.

Starvation induces a loss in the mass of the liver, which gradually decreases as the days of starvation increase^25,74^. As liver mass decreases, concentration of some metals (i.e. Cu, Zn) increases, but the total content of metals in the liver remains constant^75^. This is important because laboratory experiments using rats demonstrate that animals that are lean before undergoing starvation/fasting achieve a lower final liver protein mass before death (and die sooner) than animals that were fat before the starvation event^67^. If this same mechanism occurs in prions, we might expect prions that were lean/sub-lethally malnourished before the storm to have smaller livers and higher concentrations of some metals (especially those that are not environmentally limited) than fat/healthy prions, although this is yet to be confirmed experimentally in birds. We did not weigh total liver mass, but the increased Cu/Zn concentration observed in slender-billed prions with ingested plastic may indicate transfer, or poorer average body condition than in non-plastic eaters that died during the storm. Plastic-ingesting individuals of both species had lower liver Fe and Al concentrations than those that did not ingest plastic. Fe is a limiting nutrient in the Southern Ocean^76–78^, while Mn and Co exist at trace concentrations^79^. As Fe showed the strongest plastic-metal relationship (slender-billed prions), we suggest that the largest potential sub-lethal nutritional consequence of plastic ingestion may concern nutrients that are already environmentally limited, as is the case for Fe in the Southern Ocean^76–78^. Reduced food intake resulting from dietary dilution by plastic ingestion may lead to lower liver Fe concentration as food reduction decreases the number of red blood cells in seabirds^62^ and lower dietary Al, Mn and Co. We propose that individuals that are already sub-lethally malnourished may have a higher risk of dying than those in good condition when the extreme weather event begins, as observed in other bird species^53^.

Previous studies raise concern regarding the potential transfer of Cd and Pb between plastic and wildlife^14,31,35,39^, including plastic-mediated transfer of Cd observed in fish^14^ and suggestions that plastics may be responsible for between 6 and 30% of a seabird's exposure to and accumulation of Pb^41^. We found no significant relationships in this study that support these predictions. The estimation of plastic accounting for 6–30% of seabirds’ Pb exposure is too high for fairy and slender-billed prions collected in Australasia. Individual birds varied greatly in their Cd and Pb liver concentration (Table 1). Low Fe concentration has been linked with high uptake of Pb^80^, which presents further concern for plastic-ingesting birds with reduced Fe. This may occur because divalent metal transporter 1, which mediates the absorption of divalent cations in the small intestine, exhibits a substrate preference for Fe^80^. When the diet is deficient in Fe, absorption of Pb may increase via the divalent metal transporter 1 in the small intestinal cells^81^. If Pd and Cd transfer between plastic and body occurs, we suggest that either the magnitude is minimal and dwarfed by diet and environmental exposure, or that these metals are being detoxified in the body by binding to metallothionein^32,36,64^. If heavy metal transfer from plastic occurs, it is possible that increases may be masked by metallothionein synthesis, which would result in increases in Zn, as observed. Whether metallothionein synthesis is the cause of the increased Zn concentration with plastic presence observed, however, cannot be tested with this study.

Although the plastic load-metal(loid) concentration effect is small, the plastic ingestion dietary dilution effect may reduce survival across a large population affected by extreme weather, especially where ingested large plastic loads occur. We do not know pre-storm health, but it is likely that examined birds included individuals that were in healthy condition prior to the storm and starved rapidly under challenging conditions. Tens to hundreds of thousands of prions probably died during this single storm event, including birds in healthy initial condition, as birds can starve rapidly in prolonged extreme conditions such as high wind, rain and rough seas^53^. We have provided discussion justifying why we believe these results can be applicable to healthy prions. However, given that all bird examined were in a starved condition on collection, we do not know with certainty whether these results can be applied to healthy birds, nor is it possible to measure the plastic loads or liver metal(loid) concentrations of birds that survived the storm event. Given the variation in liver metal(loid) concentration due to other factors such as age, foraging and diet, further sampling is recommended to elucidate the relationships between ingested plastic and tissue metal(loid)s, and influence on survival of environmental challenges.

Prions are moderate plastic eaters relative to other Procellariiforme species^82–84^, and the maximum plastic loads found here are smaller than the maximum loads found among beach-cast fairy prions and slender-billed prions in another study (Fairy prions; this study max = 2, Roman et al. 2019 max = 14. Slender-billed prions; this study max = 15, Roman et al. 2019 max = 22.)^82^. If large plastic loads cause malnourishment in seabirds, it is unlikely that sub-lethally malnourished birds that die during storms will have plastic loads as large as beach-cast birds that may have died from lethal malnourishment (Fig. 1). As significant relationships between plastic ingestion and metal concentration were found in slender-billed prions, we recommend a careful examination of potential relationships in seabirds with higher rates and ingested loads of plastic, in particular fulmarine petrels^85,86^ and storm-petrels^82,87^. As seabird wreck events can lead to high mortality, the potential effect of small variations in health/condition associated with ingested plastic may push birds closer to an energetic knife’s edge. For large seabird wreck events, the effect of widespread plastic ingestion through a population on the overall death toll may not be inconsequential.

## Conclusion

Plastic ingestion in prions, especially for those individuals with large plastic loads, is associated with reduced Al, Mn, Fe and Co concentrations and increased Cu and Zn concentration in the liver. We posit that this result may be caused by dietary dilution and subsequent malnutrition due to plastic in the gut, and potentially by the transfer of Zn from ingested plastic. This new evidence shows a relationship between plastic and liver metal(loid) concentration in free-living wildlife, with a potential nutrition and pollution link. However, the effect is small and is less important than the effect of external factors such as diet and foraging differences. Plastic load, type of diet and resultant chronic dietary dilution are factors that may affect seabird body condition and consequent resilience and survival in natural events such as storms. We conclude that ingested plastics in loads such as those we examined may affect essential metal(loid) mineral nutrient absorption through dietary dilution but may not be important vectors of non-essential metal(loid) contamination in fairy and slender-billed prions.

## Supplementary information

Supplementary Information
