# Supplementary material for: Plastic, nutrition and pollution; relationships between ingested plastic and metal concentrations in the livers of two Pachyptila seabirds
Source: Sci Rep. 2020 Oct 22;10:18023. doi: 10.1038/s41598-020-75024-6 (PMC7582968; doi:10.1038/s41598-020-75024-6)

**Supplementary information for:**

**Plastic, nutrition and pollution; Relationships between ingested plastic and metal concentrations in the livers of two *Pachyptila* seabirds**

Lauren Roman ^a,b,*^, Farzana Kastury ^c^, Sophie Petit ^d^, Rina Aleman ^d^, Chris Wilcox ^a^, Britta Denise Hardesty ^a^, Mark A. Hindell ^b,e^

^a^ CSIRO Oceans and Atmosphere, Hobart, Tasmania, Australia,

^b^ Institute for Marine and Antarctic Studies, University of Tasmania, Hobart, Tasmania, Australia,

^c^ Future Industries Institute, University of South Australia, South Australia, Australia,

^d^ School of Natural and Built Environments, University of South Australia, South Australia, Australia,

^e^ Antarctic Climate and Ecosystems CRC, University of Tasmania, Hobart, Tasmania, Australia,

* [lauren.roman@utas.edu.au](mailto:lauren.roman@utas.edu.au)

Table S1: Detailed description of the plastic ingested by prions in this study

| Species | Bird ID. | Item letter | Item colour | Item type | size class | length (mm) | width (mm) | depth (mm) | mass (mg) |
| --- | --- | --- | --- | --- | --- | --- | --- | --- | --- |
| Fairy prion | 38 | a | white | fragment | Mesoplastic | 6.2 | 3.2 | 0.7 | 8.2 |
|  | 39 | a | white | fragment | Mesoplastic | 5.9 | 5.5 | 1.3 | 31.7 |
|  |  | b | white | fragment | Mesoplastic | 6.4 | 4.8 | 1.6 | 33.4 |
|  | 57 | a | grey | fragment | Microplastic | 2.7 | 2.3 | 1.9 | 7 |
|  |  | b | white | pellet | Microplastic | 3.5 | 3.5 | 2.4 | 17.8 |
|  | 59 | a | white | fragment | Mesoplastic | 6.2 | 4.2 | 0.7 | 12.6 |
|  | 61 | a | black | other (rubber) | Mesoplastic | 10.1 | 6.7 | 1.3 | 35.7 |
|  | 64 | a | white | fragment | Mesoplastic | 6.5 | 3.8 | 0.6 | 10.6 |
|  | 65 | a | black | pellet | Mesoplastic | 5.2 | 4.8 | 1.7 | 25.1 |
| Slender-billed prion | 92a  92b | a | white | fragment | Microplastic | 3 | 2.4 | 0.4 | 2.4 |
|  |  | b | white | fragment | Microplastic | 3.3 | 2.6 | 0.6 | 4.7 |
|  |  | c | white | fragment | Microplastic | 3.8 | 1.8 | 0.4 | 2.3 |
|  |  | d | white | fragment | Microplastic | 2.8 | 1.1 | 0.5 | 1.2 |
|  |  | e | white | fragment | Microplastic | 2.4 | 1.9 | 0.1 | 0.7 |
|  |  | f | green | fragment | Microplastic | 2.3 | 0.4 | 0.4 | 0.1 |
|  |  | g | red | fragment | Microplastic | 1.2 | 1 | 0.8 | 0.5 |
|  |  | a | green | fragment | Mesoplastic | 6.2 | 1.4 | 1 | 4.4 |
|  |  | b | white | fragment | Mesoplastic | 10.1 | 6.8 | 1.3 | 56.4 |
|  |  | c | green | fragment | Mesoplastic | 8.6 | 3.8 | 0.7 | 14.2 |
|  |  | d | white | fragment | Mesoplastic | 5.9 | 4.7 | 0.3 | 5.6 |
|  |  | e | white | fragment | Mesoplastic | 5.8 | 3.7 | 0.7 | 5.2 |
|  |  | f | black | fragment | Mesoplastic | 6.7 | 3.6 | 0.3 | 1.9 |
|  |  | g | white | fragment | Mesoplastic | 5 | 4.2 | 2 | 20.9 |
|  |  | h | white | fragment | Mesoplastic | 7.4 | 4.4 | 0.7 | 9.5 |
|  |  | i | grey | fragment | Mesoplastic | 5.5 | 3.8 | 1.1 | 12.8 |
| Slender-billed prion |  | j | white | fragment | Mesoplastic | 5.7 | 4.5 | 0.5 | 5 |
|  |  | k | green | fragment | Microplastic | 1.6 | 1.4 | 1 | 0.9 |
|  |  | l | blue | fragment | Microplastic | 2.2 | 1.4 | 0.8 | 1.1 |
|  |  | m | green | fragment | Microplastic | 3.1 | 2.2 | 0.6 | 1.6 |
|  |  | n | black | fragment | Microplastic | 3.1 | 1.1 | 0.5 | 1.7 |
|  |  | o | red | fragment | Microplastic | 1.5 | 0.9 | 0.4 | 0.2 |
|  | 94 | a | black | fragment | Microplastic | 4.3 | 3.5 | 0.6 | 4.7 |
|  |  | b | clear | fragment | Mesoplastic | 5 | 4.7 | 0.6 | 9.5 |
|  |  | c | clear | fragment | Microplastic | 4.7 | 4.2 | 0.4 | 5.2 |
|  |  | d | clear | fragment | Mesoplastic | 7.6 | 3.5 | 0.4 | 9.9 |
|  |  | e | brown | fragment | Microplastic | 4.9 | 3 | 0.8 | 9.9 |
|  |  | f | black | fragment | Mesoplastic | 7.4 | 4.5 | 0.2 | 8 |
|  |  | g | brown | pellet | Microplastic | 3.9 | 2.7 | 1.8 | 14.9 |
|  |  | h | brown | fragment | Mesoplastic | 5.3 | 4 | 0.5 | 8.3 |
|  |  | i | black | pellet | Microplastic | 3 | 2.8 | 2.2 | 13 |
|  |  | j | brown | pellet | Microplastic | 4.4 | 3.2 | 1.5 | 15.8 |
|  | 95 | a | blue | other | Mesoplastic |  |  |  |  |
|  |  | b | brown | linear | Mesoplastic |  |  |  |  |
|  |  | c | brown | fragment | Mesoplastic | 6 | 3.1 | 0.2 | 4.2 |
|  |  | d | white | fragment | Microplastic | 2.4 | 1.4 | 0.3 | 1.2 |
|  |  | e | white | fragment | Mesoplastic | 6.2 | 3 | 1 | 7.6 |
|  |  | f | brown | fragment | Mesoplastic | 6 | 3.8 | 0.6 | 13.9 |
|  |  | g | pink | fragment | Microplastic | 3.8 | 2.7 | 1.1 | 7.4 |
|  | 96 | a | white | fragment | Microplastic | 3.1 | 2.9 | 0.7 | 4.1 |
|  |  | b | black | pellet | Microplastic | 3.1 | 2.5 | 2.2 | 4 |
|  |  | c | black | pellet | Microplastic | 4.5 | 4.1 | 1.8 | 21.7 |
|  | 98 | a | black | pellet | Microplastic | 3.6 | 3.3 | 2.7 | 20 |
|  | 99 | a | blue | other (balloon) | Mesoplastic |  |  |  | 247.3 |
| Slender-billed prion |  | b | white | fragment | Mesoplastic | 9.7 | 8.8 | 5.2 | 121.4 |
|  |  | c | white | fragment | Mesoplastic | 7.5 | 5.4 | 1.7 | 43.8 |
|  |  | d | brown | fragment | Mesoplastic | 5 | 3.9 | 0.5 | 7 |
|  |  | e | clear | fragment | Microplastic | 3.9 | 2.9 | 0.4 | 2.4 |
|  | 100 | a | black | fragment | Mesoplastic | 5.1 | 4.6 | 0.8 | 11.5 |
|  |  | b | black | fragment | Microplastic | 4.7 | 4.6 | 0.3 | 6 |
|  |  | c | white | fragment | Microplastic | 4.1 | 4 | 0.5 | 6 |
|  | 101 | a | black | linear | Mesoplastic |  |  |  |  |
|  |  | b | white | fragment | Mesoplastic | 9.2 | 4.8 | 0.6 | 24.5 |
|  |  | c | white | fragment | Microplastic | 3.3 | 2.1 | 0.4 | 3.2 |
|  |  | d | white | fragment | Microplastic | 4.1 | 2.6 | 0.2 | 3.4 |
|  |  | e | clear | sheet plastic | Microplastic |  |  |  | 1 |
|  | 103 | a | brown | fragment | Mesoplastic | 6.3 | 4.7 | 1.2 | 10.5 |
|  | 104 | a | brown | pellet | Microplastic | 3.9 | 3.9 | 2.7 | 24.8 |
|  | 105 | a | white | fragment | Microplastic | 4.1 | 3.9 | 0.7 | 12 |
|  |  | b | white | fragment | Microplastic | 3.3 | 2.7 | 0.5 | 4.6 |
|  |  | c | brown | fragment | Microplastic | 4.5 | 2.3 | 1 | 7 |
|  | 106 | a | white | fragment | Mesoplastic | 7.8 | 4.4 | 1.1 | 25.1 |
|  |  | b | white | fragment | Microplastic | 3.1 | 2.2 | 1.2 | 5.5 |
|  |  | c | black | fragment | Microplastic | 2.1 | 1.3 | 0.2 | 0.1 |
|  | 107 | a | white | fragment | Mesoplastic | 7 | 3.3 | 0.7 | 9.3 |
|  |  | b | brown | fragment | Mesoplastic | 7.2 | 5.1 | 0.4 | 12.7 |
|  |  | c | white | fragment | Mesoplastic | 5.1 | 4.8 | 1.3 | 21.1 |
|  |  | d | brown | fragment | Mesoplastic | 8 | 4.8 | 1.1 | 25.9 |
|  |  | e | white | fragment | Mesoplastic | 6.4 | 4 | 0.2 | 9 |
|  |  | f | grey | fragment | Microplastic | 3.7 | 3.7 | 0.8 | 8.2 |
|  |  | g | white | fragment | Mesoplastic | 7 | 5.9 | 0.5 | 17.2 |
|  |  | h | brown | fragment | Microplastic | 4.6 | 5.1 | 0.6 | 7.1 |
|  |  | i | white | fragment | Microplastic | 3.3 | 1.5 | 1.4 | 3.1 |

Table S2: Detailed description of the morphometrics of the prions in this study. Please note that mass is not available for all birds as some birds were not weighed due to waterlogged and sandy condition. Age (A = adult, I = immature) and sex were not able to be assessed for some individuals.

| Bird No. | Species | Age | Sex | Plastic Ingest? | No. of ingested items | Mass of ingested items (mg) | Wing cord (mm) | Tarsus length (mm) | Head length (mm) | Culmen length (mm) | Mass (g) | Pect. muscle score | Subcut. fat score | Intest. fat score |
| --- | --- | --- | --- | --- | --- | --- | --- | --- | --- | --- | --- | --- | --- | --- |
| 46 | Fairy prion | A | f | no | 0 | 0 | 184 | 30.4 | 52.3 | 22.8 |  | 0 | 0 | 0 |
| 42 |  | A | f | no | 0 | 0 | 178 | 33.5 | 53 | 22.2 | 91 | 0 | 0 | 0 |
| 52 |  | A | m | no | 0 | 0 | 177 | 31.7 | 57.3 | 24.7 |  | 0 | 0 | 0 |
| 62 |  | A | m | no | 0 | 0 | 189 | 32.8 | 58.3 | 23 |  | 0 | 0 | 0 |
| 60 |  | A | m | no | 0 | 0 | 182 | 31.3 | 54.3 | 21.5 | 85 | 0 | 0 | 0 |
| 37 |  | A | f | no | 0 | 0 | 184 | 31.4 | 55.2 | 24.5 | 82 | 0 | 0 | 0 |
| 50 |  | A | f | no | 0 | 0 | 184 | 30.6 | 55.1 | 23.6 |  | 0 | 0 | 0 |
| 70 |  | A | m | no | 0 | 0 | 181 | 31.9 | 56.1 | 23.6 |  | 0 | 0 | 0 |
| 45 |  | A | f | no | 0 | 0 | 188 | 32.9 | 56.8 | 24.7 |  | 0 | 0 | 0 |
| 73 |  | A | m | no | 0 | 0 | 174 | 32.4 | 55.3 | 23.6 |  | 0 | 0 | 0 |
| 68 |  | A | f | no | 0 | 0 | 188 | 30 | 54.3 | 23.6 |  | 0 | 0 | 0 |
| 71 |  | I | m | no | 0 | 0 | 183 | 32.4 | 56.2 | 23.3 |  | 0 | 0 | 0 |
| 54 |  | I | m | no | 0 | 0 | 192 | 33.9 | 58.1 | 24.3 | 102 | 0 | 0 | 0 |
| 66 |  | I | m | no | 0 | 0 | 176 | 31.8 | 56.6 | 25.5 |  | 0 | 0 | 0 |
| 56 |  | I | m | no | 0 | 0 | 186 | 32.8 | 57.6 | 25.1 |  | 0 | 0 | 0 |
| 69 |  | I | m | no | 0 | 0 | 170 | 31 | 54.8 | 23.9 |  | 0 | 0 | 0 |
| 53 |  |  |  | no | 0 | 0 | 183 | 33.1 | 58.5 | 26.6 |  | 0 | 0 | 0 |
| 51 |  |  |  | no | 0 | 0 | 181 | 31.6 | 55.4 | 22.8 | 95 | 0 | 0 | 0 |
| 47 |  |  |  | no | 0 | 0 | 184 | 34.6 | 57.1 | 25 |  | 0 | 0 | 0 |
| 59 |  | A | f | yes | 1 | 12.6 | 179 | 31.8 | 56 | 22.2 |  | 0 | 0 | 0 |
| 61 |  | A | f | yes | 1 | 35.7 | 183 | 29.5 | 55.3 | 23.7 | 102 | 0 | 0 | 0 |
| 39 |  | A | f | yes | 2 | 65.1 | 176 | 32.8 | 55 |  |  | 0 | 0 | 0 |
| 65 |  | A | m | yes | 1 | 25.1 | 173 | 30.5 | 53.2 | 22 | 64 | 0 | 0 | 0 |
| 38 |  | A | m | yes | 1 | 8.2 | 180 | 31.7 | 55.3 | 24.4 | 91 | 0 | 0 | 0 |
| 57 |  | A | m | yes | 2 | 24.8 | 180 | 32.7 | 55.5 | 22.7 |  | 0 | 0 | 0 |
| 64 |  | I | m | yes | 1 | 10.6 | 183 | 32.6 | 55.7 | 23.7 | 88 | 0 | 0 | 0 |
| 85 | Slender-billed prion | A | m | no | 0 | 0 | 192 | 33.9 | 63 | 26.9 |  | 0 | 0 | 0 |
| 74 |  | A | m | no | 0 | 0 | 189 | 33.6 | 60 | 25.4 | 93 | 0 | 0 | 0 |
| 88 |  | I | m | no | 0 | 0 | 180 | 31.5 | 61.2 | 28.4 |  | 0 | 0 | 0 |
| 93 |  | I | m | no | 0 | 0 | 182 | 31.8 | 63.8 | 29.4 |  | 0 | 0 | 0 |
| 84 |  | I |  | no | 0 | 0 | 188 | 31.4 | 61.6 | 25.7 |  | 0 | 0 | 0 |
| 79 |  | I | m | no | 0 | 0 | 174 | 29.8 | 61.5 | 25.2 | 115 | 0 | 0 | 0 |
| 108 |  | I | m | no | 0 | 0 | 176 | 30.9 | 56.7 | 24.9 |  | 0 | 0 | 0 |
| 97 |  |  |  | no | 0 | 0 | 180 | 31.4 | 56.9 | 25 | 89 | 0 | 0 | 0 |
| 102 |  |  |  | no | 0 | 0 | 177 | 32.7 | 60.6 | 26.1 |  | 0 | 0 | 0 |
| 81 |  |  |  | no | 0 | 0 | 181 | 32.3 | 61 | 26 |  | 0 | 0 | 0 |
| 82 |  |  |  | no | 0 | 0 | 187 | 32.1 | 60 | 25.9 |  | 0 | 0 | 0 |
| 96 |  | I | m | yes | 3 | 29.8 | 178 | 30.8 | 59.8 | 27.9 | 77 | 0 | 0 | 0 |
| 94 |  | I | m | yes | 10 | 99.2 | 175 | 30.5 | 58.7 | 25.9 | 79 | 0 | 0 | 0 |
| 103 |  | I | m | yes | 1 | 10.5 | 175 | 32.6 | 63.6 | 26.6 |  | 0 | 0 | 0 |
| 92 |  | I | m | yes | 15 | 153.3 | 173 | 32.9 | 61 | 26.1 |  | 0 | 0 | 0 |
| 106 |  | I | m | yes | 3 | 30.7 | 187 | 32.8 | 63.7 | 27.7 | 91 | 0 | 0 | 0 |
| 99 |  | I | m | yes | 5 | 421.9 | 181 | 31.1 | 63.2 | 26.5 |  | 0 | 0 | 0 |
| 98 |  | I | m | yes | 1 | 20 | 179 | 33.2 | 61.6 | 25.1 |  | 0 | 0 | 0 |
| 96 |  | I | m | yes | 3 | 29.8 | 178 | 30.8 | 59.8 | 27.9 | 77 | 0 | 0 | 0 |
| 107 |  | I | m | yes | 9 | 113.6 | 174 | 31.6 | 62.4 | 27.2 |  | 0 | 0 | 0 |
| 100 |  |  |  | yes | 3 | 23.5 | 183 | 32.7 | 61.8 | 26.5 |  | 0 | 0 | 0 |
| 95 |  |  |  | yes | 7 | 34.3 | 176 | 31.9 | 60.3 | 27.2 |  | 0 | 0 | 0 |
| 104 |  |  |  | yes | 1 | 24.8 | 181 | 32.8 | 61.1 | 28.1 |  | 0 | 0 | 0 |
| 101 |  |  |  | yes | 5 | 32.1 | 179 | 33.5 | 61.7 | 27.5 |  | 0 | 0 | 0 |
| 105 |  |  |  | yes | 3 | 23.6 | 187 | 30.8 | 59.6 | 25.9 | 98 | 0 | 0 | 0 |

**Photos of plastic items**

Fairy prion

Bird 38


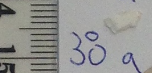


Bird 39


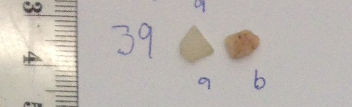


Bird 57


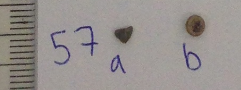


Bird 59


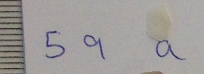


Bird 61


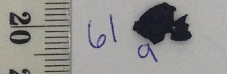


Bird 64


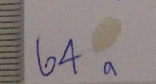


Bird 65


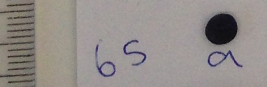


Slender-billed prion

Bird 92 (e is one item broken during handling)


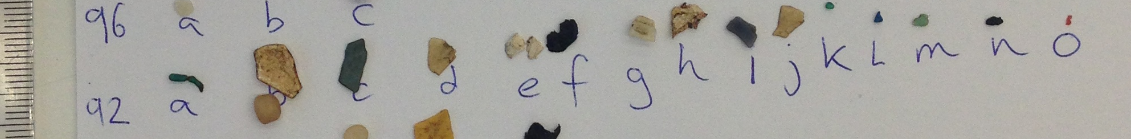


Bird 94


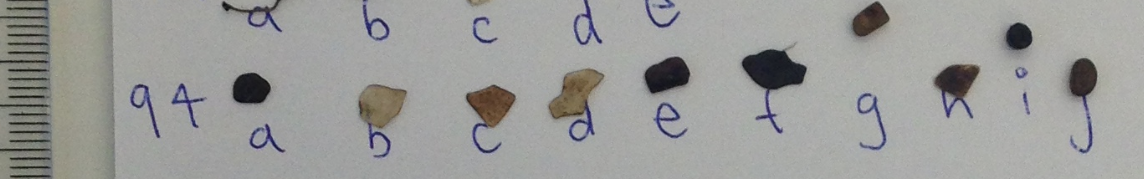


Bird 95


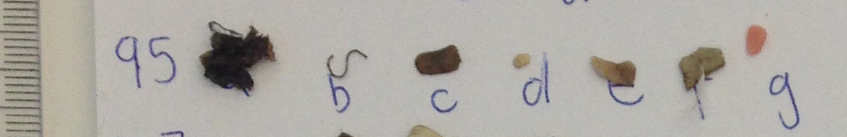


Bird 96


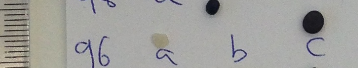


Bird 98


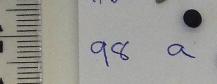


Bird 99


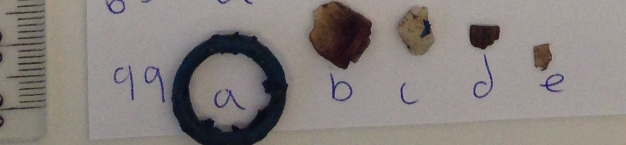


Bird 100


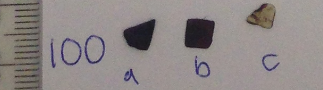


Bird 101


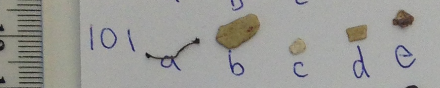


Bird 103


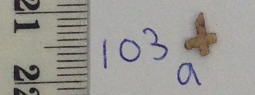


Bird 104


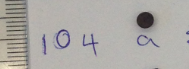


Bird 105


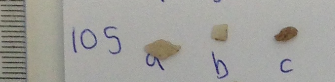


Bird 106


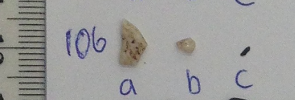


Bird 107


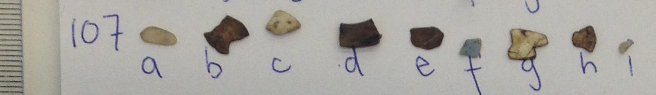


Photo of plastic fragments retained in the gizzard of a beach-wrecked fairy prion*. Note: This individual was not included in this study.*


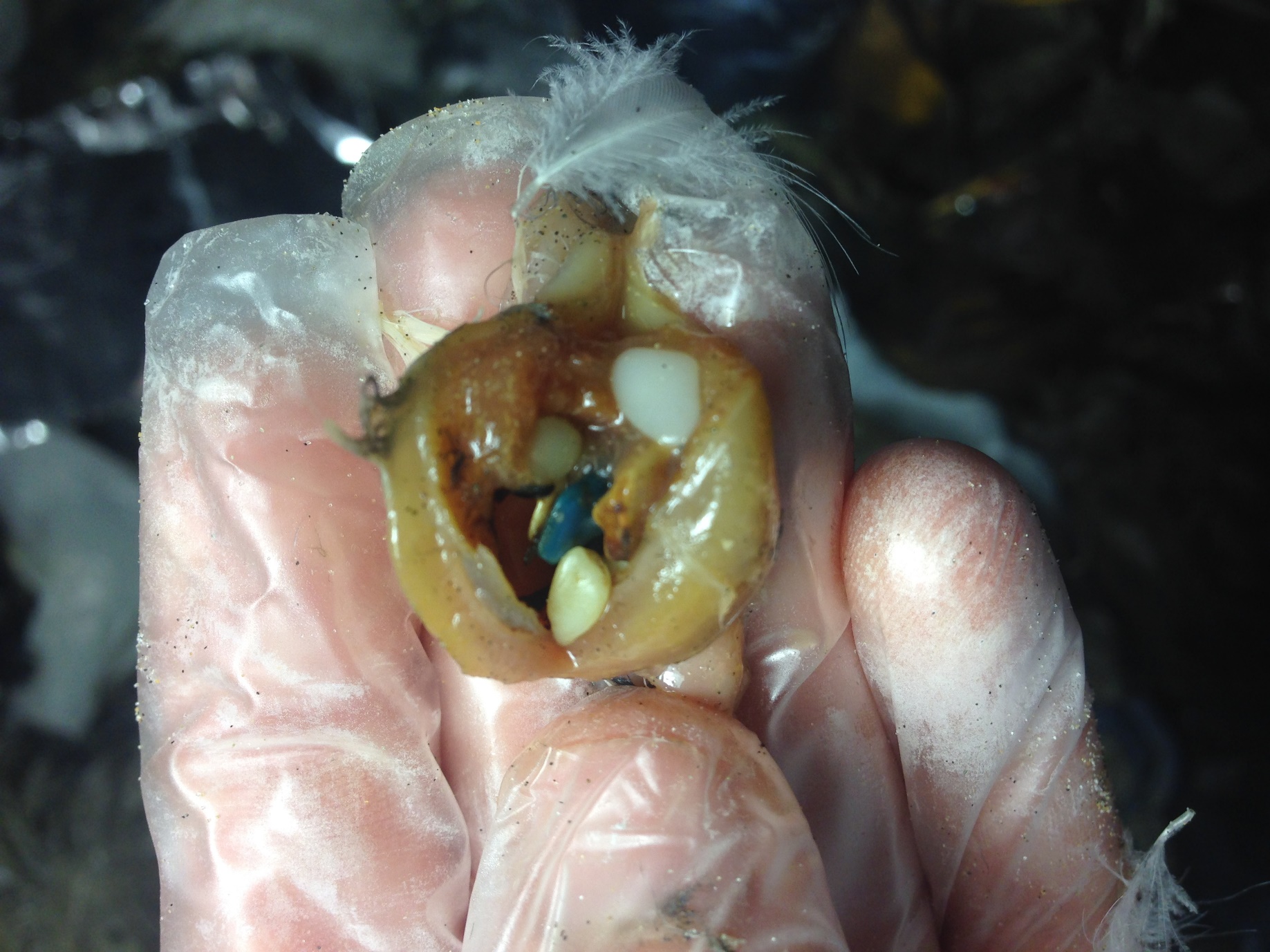

Supplement: Supplementary file 1 — Supplementary Information [file 41598_2020_75024_MOESM1_ESM.docx]
